# Penalties to Which Medical Men Are Exposed in the Discharge of Their Duties

**Published:** 1853

**Authors:** 


*Penalties to which Medical Men are exposed in the discharge of their Duties.*

Judges, including justices of the peace, are protected in the discharge of their duties; counsel are not responsible for the opinions they deliver, or for the proceedings they advise, and jurymen incur no penalty by returning verdicts even contrary to evidence. But the medical profession enjoys no such immunity. Medical men, for acts done in the discharge of their duty, are exposed to legal penalties, and may be subjected to vexatious lawsuits on the weakest and most unfounded allegations. Of this a remarkable illustration is afforded by the case Rough *v.* Rough, Lyell, and others, tried before the Lord Justice Clerk and a jury, on the 9th of August last.

The circumstances of that case are briefly these :—

The pursuer, Rough, was brought from a distance to her mother's house in a state of insanity, in February, 1847. Dr. Lyell, of Dundee, at the request of her relatives, had several interviews with her, for the purpose of examining into her state of mind; and thereby, and also from inquiries at her relatives and others, and correspondence with the medical man who attended her previously, satisfied himself of her insanity. Accordingly, he granted a certificate, on a petition to the Sheriff of Edinburgh, for warrant to confine her, and this certificate was subsequently signed by Drs. Moir and Scott, of Musselburgh, after they had carefully examined the patient. A warrant was thereafter obtained for her confinement in an Asylum, near Musselburgh, where she was in consequence placed. She remained and was treated there down till about August, 1850. By that time she had improved in health, and was, in consequence, permitted to remove and board with a family in the village of Ormiston.

Having afterwards recovered, or at least alleging that she was sane, and had never been insane, she brought an action against her relatives and others, and also against the medical men, to recover damages from the former for having wrongously confined, or caused her to be confined, and wrongously detained, or caused her to be detained, in the asylum; and from the latter for granting their certificates without due inquiry and examination.

Drs. Lyell and Scott (Moir being dead) were compelled to defend this action, and after a costly record had been made up, issues were adjusted, and the case set down for trial—much trouble and great expenses were incurred in getting up evidence and preparing. The day of trial came. A host of counsel appeared for the parties. A jury was empanelled and the trial commenced. But so utterly groundless was the pursuer's case found to be, that, after two or three of her witnesses had been examined, her counsel gave it up altogether, and the judge had to direct the jury to return a verdict for the defenders, in respect there was no evidence to go there.

The result of this triumphant refutation of the pursuer's accusation is, that Drs. Lyell and Scott, for conscientiously certifying what they believed to be true, are amerced in a heavy bill of costs. For, although the verdict entitles them to decree for expenses, that decree will, it is believed, be utterly worthless.

Surely, for such a grievance a remedy should be provided. It is absolutely necessary that medical men should be protected for acts done in the discharge of their duties. As the law presently stands, actions may be raised against them by every half-witted creature who falls into the hands of a litigating agent, on the allegation that what they did was without due inquiry and examination. But it is plain that it must always be difficult to prove due inquiry and examination. For physicians do not generally parade their actings in cases of lunacy, or publish the infirmities of their patients to the world at large. Hence, as the slightest evidence offered by the lunatic throws the burden of proof upon the medical man, due inquiry and examination should be presumed, if malice, or conspiracy, or culpable negligence be not alleged and proved. For these are the essence of crime, and a charge against a medical man of knowingly certifying a sane man, to be a lunatic, is truly a crime. It is plain that if the prosecutor has a case, proof of malice, or conspiracy, or culpable negligence could easily be offered as evidence of the want of due inquiry and examination—so the public interest would not suffer; while it is equally plain, that if some such security be not afforded to medical men, they must very often be unduly exposed to actions as groundless, and litigations as vexatious, as this case of Rough's has been proved to be.

Monthly Journal of Medical Science, October, 1853.

